# Results of the first German external quality assessment scheme for the detection of monkeypox virus DNA

**DOI:** 10.1371/journal.pone.0285203

**Published:** 2023-04-28

**Authors:** Laura Vierbaum, Nathalie Wojtalewicz, Anne Kaufmann, Sabine Goseberg, Patricia Kaiser, Hans-Peter Grunert, Ulf Dühring, Anika Zimmermann, Annemarie Scholz, Janine Michel, Andreas Nitsche, Holger F. Rabenau, Martin Obermeier, Ingo Schellenberg, Heinz Zeichhardt, Martin Kammel

**Affiliations:** 1 INSTAND e.V., Society for Promoting Quality Assurance in Medical Laboratories, Dusseldorf, Germany; 2 GBD Gesellschaft für Biotechnologische Diagnostik mbH, Berlin, Germany; 3 IQVD GmbH, Institut für Qualitätssicherung in der Virusdiagnostik, Berlin, Germany; 4 Robert Koch-Institute, Centre for Biological Threats and Special Pathogens, Highly Pathogenic Viruses, German Consultant Laboratory for Poxviruses, Berlin, Germany; 5 Institute for Medical Virology, University Hospital, Goethe University Frankfurt, Frankfurt, Hesse, Germany; 6 Medizinisches Infektiologiezentrum Berlin, Berlin, Germany; 7 Institute of Bioanalytical Sciences, Center of Life Sciences, Anhalt University of Applied Sciences, Bernburg, Saxony-Anhalt, Germany; Sun Yat-Sen University, CHINA

## Abstract

**Background:**

In May 2022, the monkeypox virus (MPXV) spread into non-endemic countries and the global community was quick to test the lessons learned from the SARS-CoV-2 pandemic. Due to its symptomatic resemblance to other diseases, like the non-pox virus varicella zoster (chickenpox), polymerase chain reaction methods play an important role in correctly diagnosing the rash-causing pathogen. INSTAND quickly established a new external quality assessment (EQA) scheme for MPXV and orthopoxvirus (OPXV) DNA detection to assess the current performance quality of the laboratory tests.

**Methods:**

We analyzed quantitative and qualitative data of the first German EQA for MPXV and OPXV DNA detection. The survey included one negative and three MPXV-positive samples with different MPX viral loads. The threshold cycle (Ct) or other measures defining the quantification cycle (Cq) were analyzed in an assay-specific manner. A Passing Bablok fit was used to investigate the performance at laboratory level.

**Results:**

141 qualitative datasets were reported by 131 laboratories for MPXV detection and 68 qualitative datasets by 65 laboratories for OPXV detection. More than 96% of the results were correctly identified as negative and more than 97% correctly identified as positive. An analysis of the reported Ct/Cq values showed a large spread of these values of up to 12 Ct/Cq. Nevertheless, there is a good correlation of results for the different MPXV concentrations at laboratory level. Only a few quantitative results in copies/mL were reported (MPXV: N = 5; OPXV: N = 2), but the results correlated well with the concentration differences between the EQA samples, which were to a power of ten each.

**Conclusion:**

The EQA results show that laboratories performed well in detecting both MPXV and OPXV. However, Ct/Cq values should be interpreted with caution when conclusions are drawn about the viral load as long as metrological traceability is not granted.

## 1. Introduction

The global awareness of possible new emerging health threats has increased considerably since COVID, the disease caused by the SARS-CoV-2 virus, was declared a pandemic in March 2020 [1]. Therefore, the spread of the zoonotic monkeypox virus (MPXV) into non-endemic countries has been followed with great interest. MPXV was first identified in 1958 as the pathogen causing a smallpox-like disease in captive monkeys [2]. Most cases were reported in Central [3] and West Africa with two identified clades in West Africa (clade II) and in the Congo Basin (clade I) [reviewed in 4, 5]. There had been sporadic events of MPXV infections outside these endemic countries [reviewed in 5], but starting in May 2022, numerous countries in Europe and the United States of America began reporting a sudden rise in MPXV infections [6]. The first cases in Germany were reported in May 2022 [7].

On July 23, the outbreak was declared a public health emergency of international concern by the WHO [8]. Since the clinical presentation of MPX resembles other infectious disease like chickenpox, which is caused by the varicella zoster virus [9, 10], laboratory diagnostics via nucleic acid amplification testing (NAAT) including real-time or conventional polymerase chain reaction (PCR) in order to correctly diagnose the rash-causing pathogen. Unlike SARS-CoV-2, which emerged as a virus in 2019, there were already several established assays targeting MPXV/orthopoxvirus (OPXV) [summarized in 11]. Although there is currently no metrologically traceable international reference preparation (IRP) available, the U.S. National Institute of Standards and Technology (NIST) has produced a synthetic DNA research grade test material covering nine PCR targets from the MPXV genome [12]. Despite the lack of metrological traceability, this control material can offer a useful tool both for the assessment of the harmonization status and for the promotion of the harmonization process, something which we have already been able to demonstrate for SARS-CoV-2 [13].

External quality assessment (EQA) schemes, also known as proficiency tests, are useful tools for evaluating the current quality of laboratory diagnostic testing. The Society for Promoting Quality Assurance in Medical Laboratories e.V. (INSTAND) has been designated as a German reference institution for quality assurance in medical laboratories by the German Medical Association. In September 2022, INSTAND became one of the first institutions worldwide to introduce an EQA scheme for MPXV and OPXV DNA detection.

In this paper, we present the first qualitative and quantitative results from this first EQA scheme for both MPXV and OPXV DNA detection.

## 2. Materials and methods

### 2.1 Sample materials–properties and preparation

Four samples were provided to the EQA participants (Table 1). One sample was virus negative and contained a cell culture lysate from non-infected MRC-5 cells (ATCC-CCL-171). The other three samples contained supernatants of cell cultures infected with MPXV (strain: MPXV WA, 2022 two clinical isolates pooled from two different patients) at different viral loads following a 10-fold dilution series. The MPXV was kindly provided by the Nationales Konsiliarlabor für Pockenviren, Robert Koch-Institut, Zentrum für Biologische Gefahren und Spezielle Pathogene, ZBS 1 –Hochpathogene Viren, Berlin, Germany.

**Table 1:** Sample properties.

Isolate/sample material	Sample no.	Dilution	MPXV DNA load (copies/mL) Conc. ± Standard deviation (SD)
MPXV clade II (2022 outbreak strain)	418001	1:10,000	15,830 ± 6,068
MPXV clade II (2022 outbreak strain)	418002	1:100,000	1,603 ± 633
MRC-5, cell lysate	418003	--	
MPXV clade II (2022 outbreak strain)	418004	1:1,000	159,403 ± 87,482

The MPXV was propagated under BSL3 conditions in Vero C1008 cells (ECACC Catalogue No. 85020206), which were maintained in a 5% CO_2_ atmosphere at 37°C in D-MEM Medium (Fisher Scientific) that included L-glutamine, supplemented with 10% fetal calf serum (FCS), 10x Pen/Strep und 100 μg/mL Normocin. The material containing the virus was not titrated before the cells were infected. The supernatant of the infected cell cultures was collected one day after infection, heat inactivated in a heating block under shaking (60°C, 2 h), and sonicated after 90 min. The infectivity of the pooled supernatant was determined using the TCID_50_ method in plaque forming units (PFU) and was reduced by this treatment from 2.2x10^7^ PFU/mL to 0 PFU/mL.

Finally, 1.1 mL of the materials were aliquoted in screw cap micro tubes (2.0 mL; Sarstedt, Nümbrecht, Germany) and lyophilized as recently described [13]. During the EQA survey, at least 11 randomly selected vials of each of the EQA samples were analyzed for stability and for homogeneity according to DIN EN ISO/IEC 17043:2010–05. Prior to the EQA survey, the source material and the EQA samples were tested by 3 INSTAND expert laboratories for suitability and declared qualified with regard to the specified properties. The viral loads of the samples, representing the anticipated concentration differences between the EQA samples of a power of ten, were estimated as the robust mean of all reported quantitative results (both MPXV and OPXV) using algorithm A [14, Section 3].

### 2.2 Ethics statement

For all patient-derived virus samples, the patient’s informed written consent was obtained for the project. An approval from the ethics committee of the Berlin Medical Association (Berliner Ärztekammer) with the number Eth44/22 was obtained for the MPXV clinical isolates used for the preparation of the EQA samples via cell culture procedures. All methods were carried out in accordance with relevant guidelines and regulations.

### 2.3 EQA procedure

INSTAND, accredited according to DIN EN ISO/IEC 17043:2010 [15], conducted its first EQA scheme for the detection of MPXV DNA in September 2022. The survey consisted of four EQA samples (see Section 2.1).

The lyophilized samples had to be reconstituted with 1.1 mL double distilled water (sterile, pyrogen-free, PCR-grade) for 20 minutes at room temperature. Laboratories could report their qualitative and quantitative results for both MPXV DNA and OPXV DNA back to INSTAND via the RV-Online platform (http://rv-online.instandev.de), including detailed information on the test system(s) used for each analysis, such as test kit supplier(s) and test kit(s) (S1 Table). Multiple results for each sample, obtained by different test systems, could be entered. A short summary of the different methods can be found in Table 2.

**Table 2:** Aggregated information provided by the participating laboratories on their methods and target genes used for all analysis combined (qualitative and quantitative for MPXV and OPXV).

**a) Extraction method**	**No. of analyses**	**d) Gene region**	**No. of analyses**
Magnetic particle technology	94	J2L/J2R	9
Silica membrane technology	54	TNF receptor	7
Extraction included in test kit	5	F3L	6
membrane based extraction	1	14 kDa gene	5
no information provided	62	E9L	5
All	216	RAP94	4
		G2R	4
**b) Detection method**	**No. of analyses**	GR2, F3L	3
Real Time PCR (TaqMan-Format)	65	rpo	3
Real Time PCR (LightCycler)	50	Hämagglutinin	3
Real Time PCR (by probes)	35	B7R	2
commercial test system	6	IMV membrane protein	2
Real Time PCR (melting courve)	3	OPG185	2
Agarose gel electrophoresis	2	17L	1
DNA Sequencing	2	A29L	1
other	2	G2R, C3L	1
Hybridization with labelled probe	1	O2L en F3L	1
no information provided	50	OPG065	1
All	216	no information provided	156
		All	216
**c) PCR method**	**No. of analyses**		
PCR / NAT	212		
digitale PCR	4		
All	216		

### 2.4 Data evaluation

For MPXV, we evaluated a total of 141 qualitative datasets provided by 131 laboratories, and 5 quantitative datasets provided by 4 laboratories. For OPXV, we evaluated a total of 68 qualitative datasets provided by 65 laboratories, and 2 quantitative datasets provided by 2 laboratories. Due to the few reported quantitative data, no further statistical analysis was performed. Nevertheless, the distribution of the quantitative data is shown for the three MPXV positive samples, in order to give a complete report of the EQA results.

An evaluation of the threshold cycle (Ct) or other measures defining the quantification cycle (Cq) was done using a test kit-specific approach for assays with at least 5 results per sample. The Grubbs’ test was performed to detect outliers in the reported Ct/Cq values, using a significance level of 0.05. Three outliers were not included in further calculations.

To check the equality of variance of the reported assay-specific Ct/Cq values per MPXV positive sample, the Levene’s test was run.

In addition, a Passing Bablok fit was performed to evaluate the dependency of the individual, laboratory-specific differences between the Ct/Cq values of the three positive samples at different concentrations.

Basic statistical analyses were performed using JMP 16.0 from SAS Institute (Cary, North Carolina, USA).

### 2.5 Generation of images

The overlay images were generated using the GIMP–GNU Image Manipulation Program 2.10.3.

## 3. Results

Our study evaluated the interlaboratory results of the qualitative and quantitative detection of MPXV and OPXV DNA from the first German 2022 EQA survey, which comprised four different samples.

**Fig 1:**
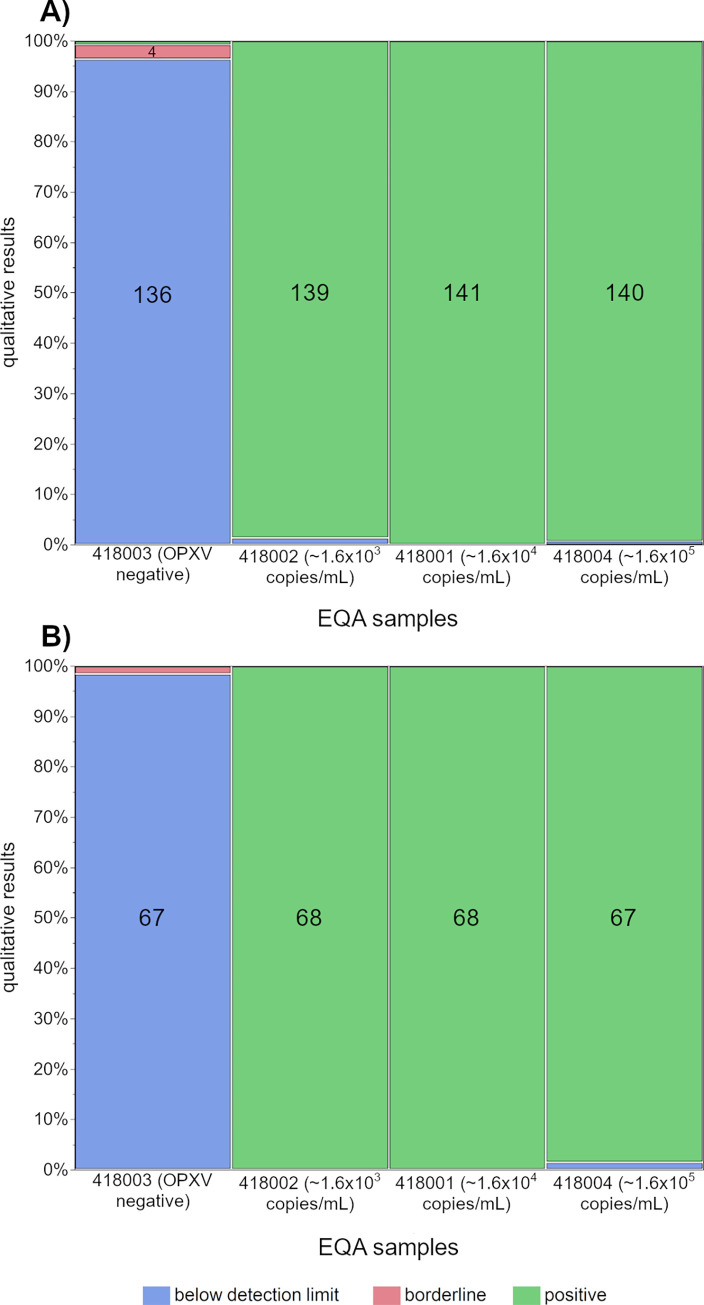
Distribution of the qualitative PCR results for the four samples of the monkeypox EQA survey for A) monkeypox virus (MPXV) and B) orthopoxvirus (OPXV). Numbers in the columns represent the actual number of results for the corresponding category.

For the negative sample containing the MRC-5 cell lysate, 136 MPXV results (96.5%) and 67 OPXV results (98.5%) were correctly identified as virus negative. Four participants reported a borderline result using an MPXV assay (2.8%) and one using an OPXV assay (1.5%); one laboratory using an MPXV assay (0.7%) identified the sample falsely as MPXV positive. Of the five false-borderline results, two cases might be due to a sample mix-up, as one participant reported the most diluted MPXV sample (~1.6 x 10^3^ copies/mL) and one participant the most concentrated OPXV sample (~1.6 x 10^5^ copies/mL) as being “below the detection limit”. The positive samples were correctly identified by nearly all participants for both analyses, with few exceptions (Fig 1, Table 3, S2 and S3 Tables).

**Table 3:** Qualitative PCR results for the four samples of monkeypox EQA survey for A) MPXV DNA and B) OPXV DNA.

**A)**					
Sample	Estimated copies/mL MPXV DNA	No. of results	N negative	N borderline	N positive
418003	Negative	141	136	4	1
418002	~1.6x10^3^	141	2	0	139
418001	~1.6x10^4^	141	0	0	141
418004	~1.6x10^5^	141	1	0	140
**B)**					
Sample	Estimated copies/mL MPXV DNA	No. of results	N negative	N borderline	N positive
418003	Negative	68	67	1	0
418002	~1.6x10^3^	68	0	0	68
418001	~1.6x10^4^	68	0	0	68
418004	~1.6x10^5^	68	1	0	67

**Fig 2:**
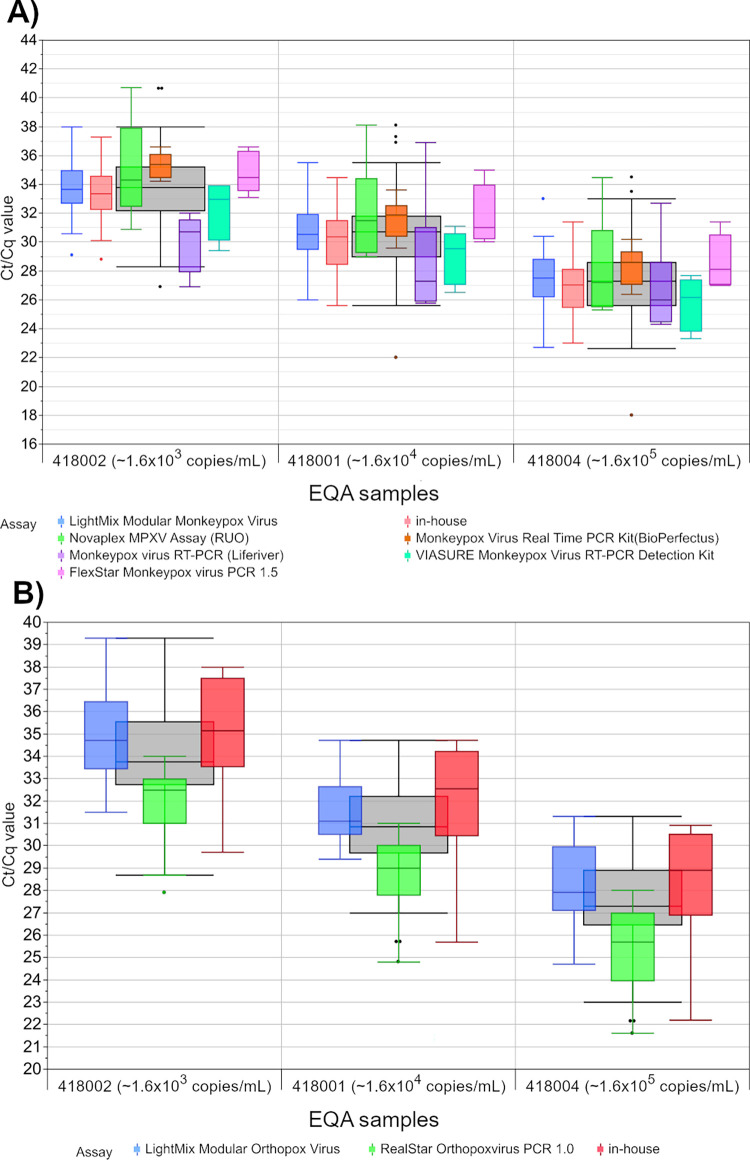
Analysis of Ct/Cq values for (A) monkeypox virus PCR results and (B) for orthopoxvirus PCR results for different test systems. The grey boxes display all results for the respective sample, and the distributions of specific manufacturer-based collectives are illustrated as smaller, colored box plots in overlay with the total results. For all boxes, the whiskers stretch from the 1st quartile—1.5*(interquartile range) to the 3rd quartile + 1.5*(interquartile range).

For 131 MPXV and 59 OPXV data sets, the participating laboratories reported their respective Ct/Cq values for the positive samples. The results for all assays with five or more results are presented in Fig 2 and Table 4. For MPXV detection, the intra-assay variability of the Ct/Cq values was between 2.4 Ct/Cq (Monkeypox Virus Real Time PCR Kit (Bio Perfectus)) and 9.8 Ct/Cq (Novaplex MPXV Assay (RUO)) for a viral load of ~1.6 x 10^3^ copies/mL, and between 3.8 Ct/Cq (Monkeypox Virus Real Time PCR Kit (Bio Perfectus)) and 10.3 Ct/Cq (LightMix Modular Monkeypox Virus) for a viral load of ~1.6 x 10^5^ copies/mL. For OPXV, the scatter of Ct/Cq values of the individual assay collectives were similar between the three different MPXV positive samples. The scatter ranged between 5.3 Ct/Cq (LightMix Modular Orthopox Virus) and 9.0 Ct/Cq (in-house), each observed for the sample with a viral load of ~1.6 x 10^4^ copies/mL.

**Table 4:** Calculation of the scatter in Ct/Cq values for the MPXV positive samples.

Isolate/sample material	Sample no.	Dilution	Ct/Cq value Mean ± SD	CV Ct/Cq value [%]
MPXV clade II (2022 outbreak strain)	418002	1:100,000	33.7 ± 2.3	6.7
MPXV clade II (2022 outbreak strain)	418001	1:10,000	30.5 ± 2.3	7.4
MPXV clade II (2022 outbreak strain)	418004	1:1,000	27.3±2.2	8.0

Since the Ct/Cq value distribution of the test collectives was compared, the variability of their variances was first tested by Levene’s test and showed overall good equality of the variances, with the exceptions of three collectives (S4 Table). The exceptions can be explained by the small collective sizes.

For MPXV detection, we observed differences between the collective medians for the individual samples, ranging from 0.6% (LightMix Modular Monkeypox Virus and in house assays for the sample containing ~1.6 x 10^3^ copies/mL) to 14.4% (Monkeypox virus RT-PCR assays from Liferiver and BioPerfectus for the sample containing ~1.6 x 10^4^ copies/mL). For OPXV detection, the assay-specific median differences were around 10%.

Nevertheless, the median values of all reported results show a clear difference of approx. 3 Ct/Cq for the three samples of the 10-fold dilution series, the value distributions are clearly overlapping even between the lowest and the highest concentrated sample for both MPXV and OPXV detection. At an assay-specific level, some assays show clearly distinguishable value distributions between the samples with the highest and lowest concentrations.

**Fig 3:**
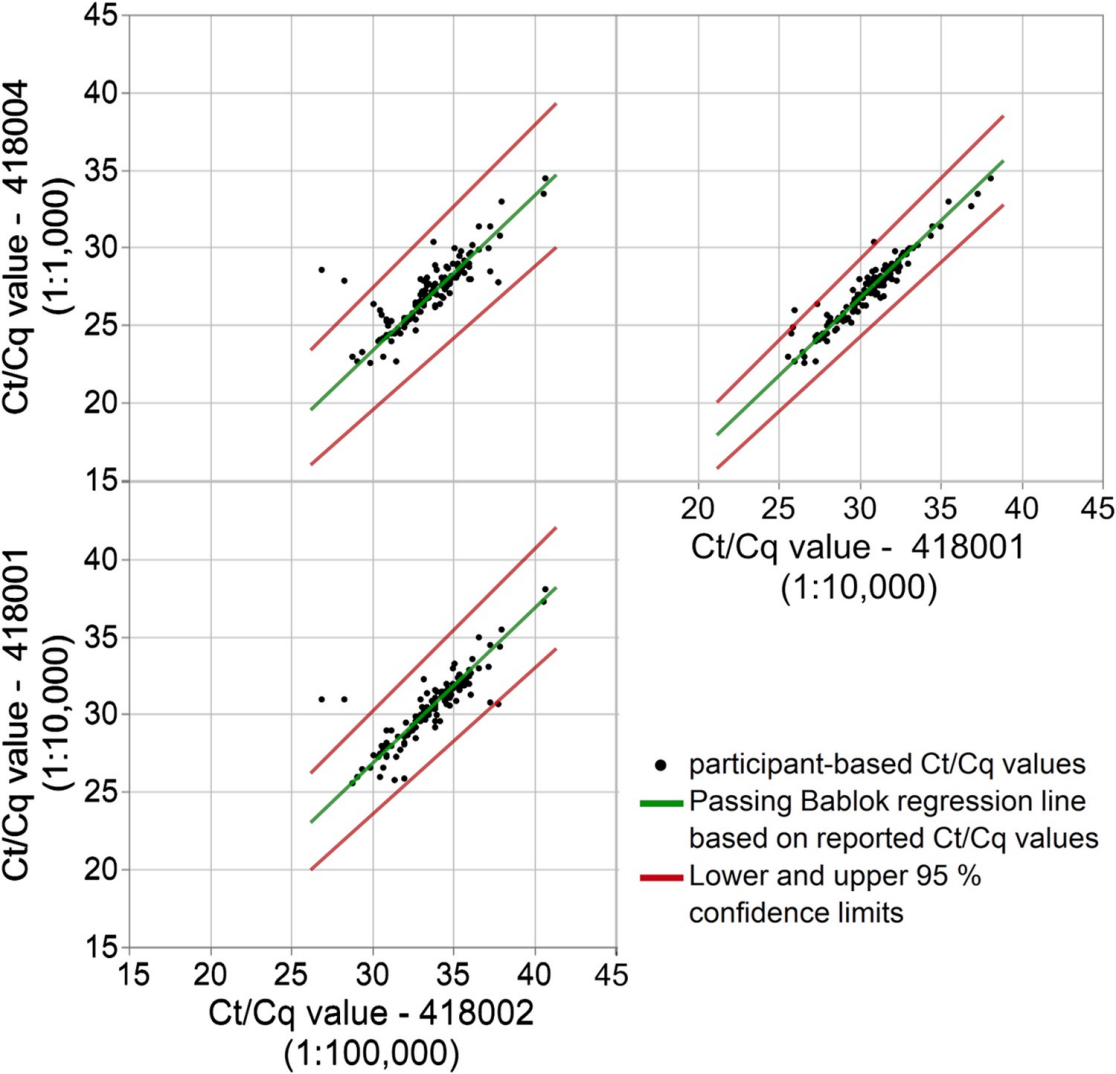
Passing Bablok fit of laboratory-based Ct/Cq values (black dots) for all pairs of EQA samples, differing in concentration by a power of ten. The Passing Bablok regression line (green) is shown with the lower and upper 95% confidence limits (red).

Passing Bablok regression analyses were performed for each sample pair to check the individual performance of the laboratories and their capability to recognize the 10-fold or 100-fold difference in MPXV viral load. For a 10-fold difference, the expected Ct/Cq-difference would be 3.32 cycles, and for a 100-fold difference it would be 6.64 cycles. The calculated Ct/Cq-differences were very close to the anticipated differences observed for all sample pairs (Fig 3, Table 5).

**Table 5:** Results of the Passing Bablok fit for the MPXV Ct/Cq results.

Compared samples	x-fold difference in MPXV viral load	Passing Bablok regression line, slope	95 % confidence limits, slope	Passing Bablok regression line, intercept	95 % confidence limits, intercept
418004–418001	10-fold	1.00	0.96–1.04	3.30	1.97–4.55
418004–418002	100-fold	1.00	0.93–1.06	6.70	4.40–8.67
418001–418002	10-fold	1.00	0.95–1.05	3.30	1.65–5.04

**Fig 4:**
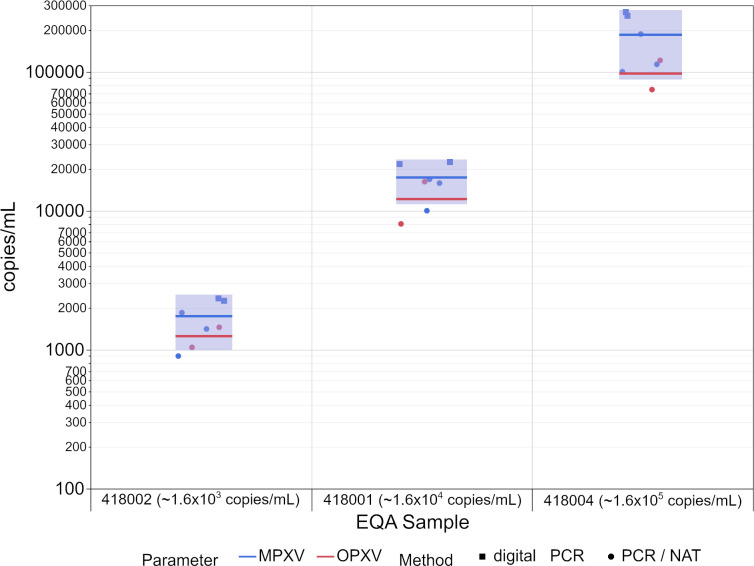
Distribution of the quantitative PCR results for monkeypox virus (blue) and orthopoxvirus (red) for the digital PCR (squares) and PCR/NAT (dots) methods. Seven participants reported results in copies/mL. The colored lines indicate the mean values of the quantitative results for MPXV (blue) and OPXV (red) respectively, and the blue area indicates the 95% confidence interval of the mean value for the quantitative MPXV PCR results. A confidence interval for OPXV was not feasible.

For the quantitative results, the mean values showed a good correlation with the different viral loads with a dilution factor of 10. Furthermore, the 95% confidence intervals of the mean value for MPXV PCR did not overlap (Fig 4, Table 1). Since there were only two quantitative results for the OPXV PCR, a calculation of the 95% confidence intervals was not feasible.

## 4. Discussion

On July 23, 2022, the WHO declared the MPXV outbreak an international public health emergency [8]. Previously, individual cases occurred only rarely outside the endemic countries in Central and West Africa. With the outbreak of the infectious disease, the number of MPXV tests increased significantly in non-endemic regions and the demand for interlaboratory comparisons was correspondingly high. Such comparisons can provide laboratories with helpful information on the quality of their (newly implemented) analytical methods and can thus help to improve the quality of (in vitro) diagnostics [16]. INSTAND, a non-profit medical society for promoting quality in medical laboratories, promptly introduced an EQA scheme in September 2022 for MPXV and OPXV DNA detection. Nucleic acid analysis by PCR is a highly sensitive and reliable technique for diagnosing pathogens [17, 18]. Several MPXV/OPXV assays were already available before the worldwide MPXV outbreak in 2022 [summarized in 11]. While an IRP for MPXV is not yet available, NIST has established a synthetic DNA research grade test material covering nine PCR targets from the MPXV genome [12] which it shares with interested laboratories to raise the current quality of in vitro diagnosis of MPXV. Data from EQA surveys are especially helpful for assessing the current status of the harmonization of the different assay results.

This paper summarizes the interlaboratory results of the first German EQA survey for the DNA detection of MPXV and OPXV for three different concentrations of MPXV positive samples (~1.6 x 10^5^ copies/mL, ~1.6 x 10^4^ copies/mL and ~1.6 x 10^3^ copies/mL), and one OPXV negative sample. 131 laboratories participated in the qualitative MPXV detection and 65 in the qualitative OPXV detection. Most of the laboratories also reported Ct/Cq values. In contrast, only a small number of laboratories reported quantitative results in copies/mL, thus the results are subject to low reliability. It should be noted that virus identification is crucial for enabling optimized patient management, consisting of isolation and therapy, since symptoms associated with MPXV are very similar to those of other infections accompanied by a febrile rash e.g. by varicella zoster virus [10].

For all EQA samples, the qualitative results for both MPXV and OPXV detection were satisfactory, as results were only occasionally incorrect (Fig 1). As laboratories only reported incorrect results for individual samples, these errors are more likely to be individual errors than to be systematic errors in internal processes or miscalibration of a laboratory device. Even for the lowest MPX viral load of ~1.6 x 10^3^ copies/mL, all participants correctly identified the positive OPXV sample and almost 100% of participants correctly identified the positive MPXV sample. False negative results using PCR technology are a general indication of the detection limit of a specific test and thus the test’s sensitivity. The inclusion of EQA samples with lower MPX viral loads in upcoming surveys will be helpful to get an impression of the different test sensitivities and the overall MPXV detection quality.

In the case of the OPXV/MPXV negative sample, only 1.5% of results (N = 1) were borderline in detecting OPXV and 2.9% of the results (N = 4) were borderline in detecting MPXV. False-positive results for MPXV are more critical, as they could result in unnecessary or even incorrect treatment of patients [19, 20]. Due to the high specificity of the test primers to the genomic target sequence of the respective virus, false positive results are unusual for PCR technology and indicate a contamination [11] or a sample mix-up. In this case, the laboratory’s internal processes have to be revised and measures for correcting errors need to be initiated. During the early stage of the international MPXV outbreak in 2022, the Centers for Disease Control and Prevention (CDC) adapted its guidance [21] in response to the treatment of falsely diagnosed patients and the post-exposure prophylaxis vaccination of their close contacts. The CDC now recommends carefully reviewing monkeypox test results from low-risk individuals and specimens with a high Ct/Cq value, and to consider other potential diagnoses, including varicella or molluscum contagiosum [22, 23]. Even though only minimal adverse effects were observed in patients that were treated with Tecovirimat [24, 25], false-positive PCR results need to be prevented, especially given the fact that MPXV might develop a resistance to the drug [26–28]. In the case of a suspected false-negative result in high-risk individuals, an additional test using another target gene should be used, since a rare tumor necrosis factor receptor gene deletion has already been reported in America [29].

Although no IRP is currently available, the quantitative EQA results in copies/mL were found to correlate well with the concentration differences by a power of ten for the different EQA samples (Fig 4). For the median Ct/Cq values, a good correlation with the different viral loads of the EQA samples was observed as well (Fig 2).

Looking at the results of the individual samples, the median Ct/Cq values of tests from different manufactures were quite well aligned and showed a difference in collective medians of < 15% for MPXV detection and < 12% for OPXV detection. When all sample and assay data are considered, a wide spread of between 2.4 Ct/Cq to 10.3 Ct/Cq was observed. Due to this high variability, it is not surprising that the manufacturer-specific data distributions clearly overlap between samples whose concentrations differ by a power of ten. This highlights the importance of the lessons previously learned from the SARS-CoV-2 pandemic that Ct/Cq values should be interpreted with caution when drawing conclusions about the viral load as long as there is no metrological traceability [13]. On a positive note, despite the overall dispersions of the Ct/Cq values for the various assays, most laboratories were able to distinguish between the different MPXV loads (Fig 3). The Passing Bablok regression lines were almost congruent with the expected relationship between samples with a 10-fold or 100-fold concentration difference (Table 5).

One limitation of our study is the use of materials of cell culture origin instead of clinical material, which could be accompanied by influencing factors such as matrix effects. This has not yet been investigated but the assumption of the commutability of the material is based on many years of experience and studies of analogously produced materials of cell culture origin. Furthermore, the source material and the EQA samples were successfully tested to be fit for purpose by three INSTAND expert laboratories.

Future EQA surveys are necessary to confirm the observed quality and reliability of the MPXV and OPXV analyses and to further monitor the current quality of MPXV and OPXV diagnostics to enable laboratories to conduct a validated interpretation of their test results. Moreover, EQA schemes containing other OPXVs, such as vaccinia and cowpoxviruses, or other rash-inducing viruses like varicella zoster, might provide additional information on the specificity of the current assays.

## Supporting information

S1 Table INSTAND Monkeypox EQA September 2022—all results. This table contains the raw results of the EQA participants without any correction. Results that were excluded from evaluation are highlighted in orange.
(XLSX)

S2 Table Tables with MPXV qualitative results submitted by EQA participants. Test kits that showed less than 5 results were aggregated into "other” (N<5). For more details about these tests, please see S1 Table. The correct result for the corresponding samples is highlighted in light grey. The quota is defined as percentage of correct results in relation to all results for both the individual test kit (horizontally) and over all test kits for the corresponding sample (sum).
(XLSX)

S3 Table Tables with OPXV qualitative results submitted by EQA participants. Samples 418001, 418002 and 418004 are positive for MPXV DNA. Test kits that showed less than 5 results were aggregated into "other (N<5). For more details about these tests, please see S1 Table. The correct result for the corresponding samples is highlighted in light grey. The quota is defined as percentage of correct results in relation to all results for both the individual test kit (horizontally) and over all test kits for the corresponding sample (sum).
(XLSX)

S4 Table Results of Levene’s test of equality of variance of the reported assay-specific Ct/Cq values per MPX positive sample for A) MPXV detection and B) OPXV detection. When equality of variance was not found (labeled in red colour), one collective (marked with *) was identified and excluded before rerun the test to be able to show the equality of the variances for the other collectives (labeled in green colour).
(XLSX)
